# Removal of Bisphenol A and Its Potential Substitutes by Biodegradation

**DOI:** 10.1007/s12010-020-03247-4

**Published:** 2020-01-20

**Authors:** Robert Frankowski, Agnieszka Zgoła-Grześkowiak, Wojciech Smułek, Tomasz Grześkowiak

**Affiliations:** 1 Institute of Chemistry and Technical Electrochemistry, Poznan University of Technology, Berdychowo 4, 60-965 Poznań, Poland; 2 Institute of Technology and Chemical Engineering, Poznan University of Technology, Berdychowo 4, 60-965 Poznań, Poland

**Keywords:** Bisphenol A, Bisphenol S, Bisphenol F, Bisphenol AF, Bisphenol E, Bisphenol B

## Abstract

The possibility of removing bisphenol A and its five potential substitutes (bisphenols S, F, AF, E, and B) was tested using microorganism consortia from river water and activated sludge from municipal and rural wastewater treatment plants. For most bisphenols, biodegradation with activated sludge was faster than with river water and a greater extent of biodegradation was also achieved. However, only bisphenol A and bisphenol F underwent 100% primary biodegradation while other bisphenols degraded no more than about 50% which has some important implications in case of their increased usage. Metabolic activity in biodegradation liquors was also tested and it showed higher activity in the tests with activated sludge than with river water. However, there was no clear connection between the decline of metabolic activity and the extent of biodegradation as decreased activity was observed for two easily degrading bisphenols and two others with little biodegradability. It can be assumed that two different phenomena are involved in this process including depletion of nutrients for easily degradable bisphenol A and absence of nutrients for bacteria incapable of primary degradation of bisphenol AF and bisphenol S.

## Introduction

**Fig. 1:**
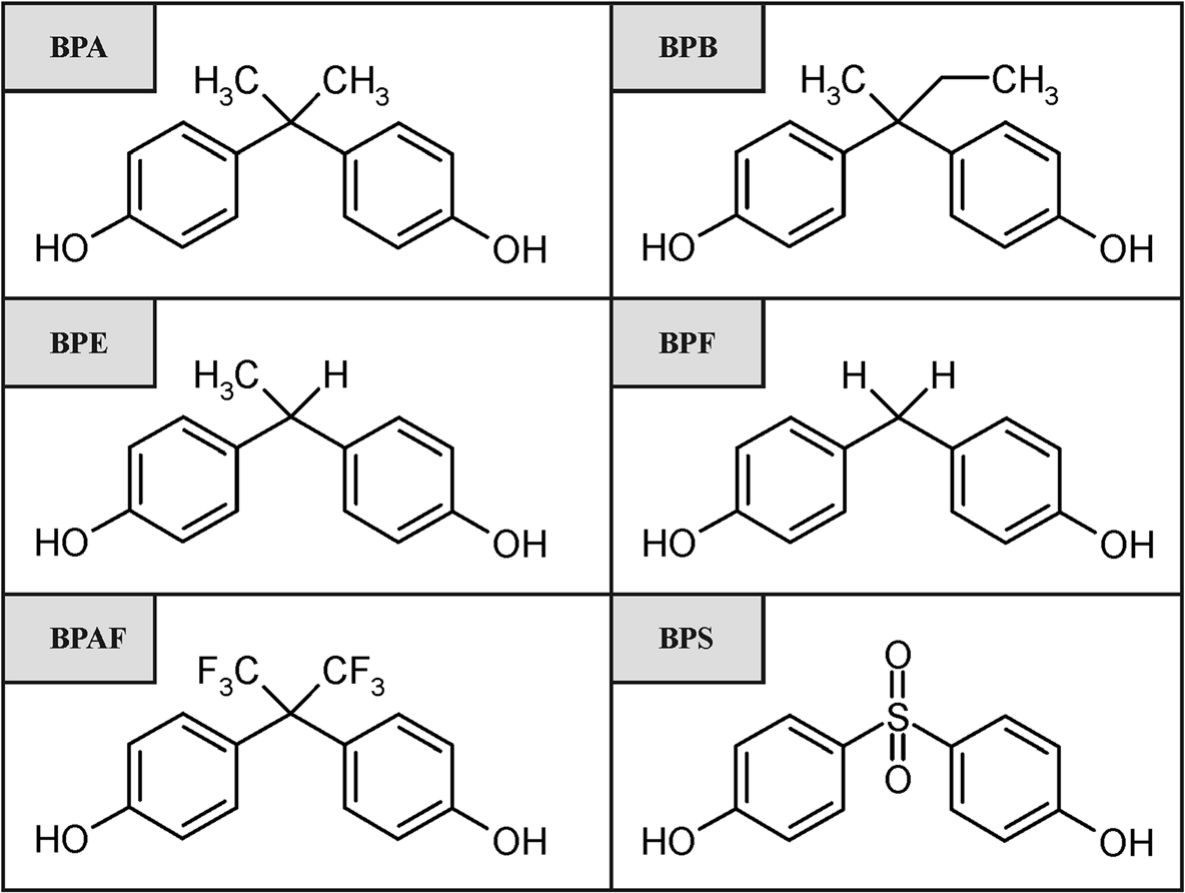
Chemical formula of bisphenols: bisphenol A (BPA), bisphenol B (BPB), bisphenol E (BPE), bisphenol F (BPF), bisphenol AF (BPAF), and bisphenol S (BPS)

Bisphenols are a group of compounds containing two phenyl rings connected to each other with a small linking group (Fig. 1). Among them, the most widely used in industry is bisphenol A (BPA), which was discovered in 1891 [1]. Annual production of BPA in 2015 was estimated at about 4.85 mln tonnes [2], and 90% of its global output is used in manufacturing of polycarbonates and epoxy-phenol resins. BPA can be found in many everyday use items such as CD/DVDs, paints, food packaging, and thermal paper commonly used in receipts at cash registers [3].

BPA belongs to the class of endocrine-disrupting compounds (EDCs) which are substances that can effectively disturb the endocrine system [4]. The first report on endocrine-disrupting properties of bisphenols included also the entire range of other organic compounds containing phenyl groups [5]. Today, a list of potential EDCs based on scientific studies contains about 1.5 thousand positions and in the last 2 years, almost 500 substances were added [6]. BPA, because of its large and widespread usage, is a great threat to the environment even though its estimated hormonal agonistic activity is much less than that of the natural hormone 17β-estradiol [7].

Human exposure to BPA is mainly related to its usage in food packaging and thermal paper [8, 9]. It can leach from the linings of food cans or from polycarbonate bottles. However, both the Food and Drug Administration (FDA) and the European Food Safety Authority (EFSA) consider levels of BPA in food as not exceeding the limits which cause adverse effects [10, 11]. Nevertheless, BPA absorption through skin by contact with receipts made of thermal paper is a major concern, and therefore the European Union has set the limit for BPA concentration in thermal paper to 0.02% by weight beginning on January 2020 [12]. This regulation will force manufacturers to replace BPA with other compounds that possess similar properties.

To date, usages of other bisphenols are infrequent. Analysis of various food packaging (e.g., baby bottles, food packaging paper, paper cups, food packaging film) revealed that BPA was a major contaminant detected in 95% of samples, ranging from 9.2 to 62.7 μg/mg while bisphenol S (BPS) was found only in one of 42 samples at 7.9 μg/mg, and both bisphenol F (BPF) and bisphenol AF (BPAF) were not detected at all [13].

In some applications, usage of BPS is relatively common because of its greater thermal stability than BPA. Monitoring of BPS throughout this millennium has found that detection frequency of BPS in human urine samples was about 20% in years 2000–2007 and about 70% in years 2009–2014 [14], which supports an increase in usage of BPS in recent years.

BPF possesses similar endocrine activity as BPA [2] but so far it is not considered as an ubiquitous contaminant. However, BPF was detected in approximately 55 of 100 urine samples with a median of 0.08 μg/L [15] and in 42% of urine samples taken from US citizens between the years 2000 and 2014 with concentrations of 0.15 μg/L to 0.54 μg/L [14].

BPAF is a BPA analog in which methyl groups are replaced by trifluoromethyl groups which can increase both chemical stability and thermal stability of the compound. It is used in the production of electronics and optical fibers, and in the processing of fluoroelastomers [16, 17]. BPAF is not yet as widely spread in the environment as BPA. It was detected in less than 3% of tested urine samples, with concentrations from < 0.1 to 0.12 μg/L [14]. Even living near a manufacturing plant does not increase the detection of BPAF in more than one-third of tested patients. The geometric mean value of BPAF in urine was 0.018 μg/L [18].

Generally, BPF, BPS, and other bisphenols appear to be the natural replacement of BPA. The similar structures of these substitutes enable their usage in common BPA applications leading to immediate compliance to different laws that forbid the use of BPA. However, the most important factor for exchanging BPA should be the reduction of releasing endocrine-disruptive compounds into the environment. This however will be difficult with bisphenols because studies have found similar disruptive activity of BPS and BPF compared with that of BPA [2, 7]. Additionally, some problems with biodegradation of these compounds have also been reported [19], which further diminish the possibility of the usage as replacements of BPA.

In the present study, biodegradation of six bisphenols (BPA, BPF, BPS, BPAF, bisphenol E (BPE), and bisphenol B (BPB)) was performed in three tests each. There were three different inocula used for each bisphenol testing: river water and activated sludge from two wastewater treatment plants (WWTPs), one from a rural area, and the other one from an urban area. According to the authors’ best knowledge, this comparison of primary biodegradation of all six bisphenols was done for the first time in the present investigation, and it clearly reveals the risk that is associated with replacement of BPA with other bisphenols.

## Materials and Methods

### Chemicals

Standards of bisphenol A, bisphenol F, bisphenol S, bisphenol AF, bisphenol E, and bisphenol B (Fig. 1) were purchased from Sigma-Aldrich (St. Louis, MO, USA) and were each of > 99% purity. MS-grade methanol was purchased from Sigma-Aldrich, and MS-grade acetonitrile was from POCh (Gliwice, Poland). As an additive to HPLC, mobile phase ammonium acetate ≥ 99% (Fluka Analytical, Switzerland) was used. The HPLC-grade water was prepared by reverse osmosis in a Demiwa system from Watek (Ledec nad Sazavou, Czech Republic), followed by double distillation from a quartz apparatus. The yellow tetrazole dye 3-(4,5-dimethylthiazol-2-yl)-2,5-diphenyltetrazolium bromide (MTT) used in the MTT assay was purchased from Sigma-Aldrich. All salts used for preparation of media were purchased from Avantor (Gliwice, Poland).

### Biodegradation Tests

The biodegradation tests were performed in duplicate separately for each bisphenol at a concentration of 10 mg/L. Each bisphenol was tested in three types of evaluation in which different inocula were used. There was river water inoculum and inocula from two WWTPs. The inoculum for the river water test was taken from the middle of the mainstream of the River Warta in Poznań (population 550,000) below the St. Roch Bridge near Poznan University of Technology campus. The river water was used in the tests directly without any pretreatment. The test was performed in 250-mL glass bottles containing 50 mL river water spiked with each bisphenol and lasted up to 52 days. The activated sludge inocula for the tests were taken from bioreactors of two WWTPs: a small rural wastewater treatment plant located in Tarnowo Podgórne which treats 3000 m^3^/day of sewage and the Left-Bank Wastewater Treatment Plant in Poznań which treats 35,000 m^3^/day of sewage. The medium used in the tests consisted of mineral components (g/L): Na_2_HPO_4_ 7.0; KH_2_PO_4_ 2.8; NaCl 0.5; NH_4_Cl 1.0 and microelements (μg/L): FeSO_4_ × 7H_2_O 0.035; MgSO_4_ × 7H_2_O 0.35; MnSO_4_ × 5H_2_O 0.2; CuSO_4_ × 7H_2_O 0.2; CoSO_4_ × 7H_2_O 0.025; H_3_BO_3_ 0.285; ZnCl 0.105, as well as yeast extract at appropriate concentration. Two series of tests were performed in 250-mL glass bottles and continued for 52 days. The tests were done using 45 mL of medium and 5 mL of activated sludge inoculum which were spiked with selected bisphenol. The samples from the tests were collected on selected days, diluted with methanol (1:3, v/v), and filtered through a 0.2-μm PTFE syringe filter to stop biodegradation. The samples were stored in a refrigerator before the LC-MS/MS analysis. Before the LC-MS/MS determination, the samples were further diluted up to 5 times to fit the linear range of the mass spectrometer. The concentrations of bisphenols determined by HPLC were used to calculate primary degradation in the tests. This was done by dividing the decrease in bisphenol concentration by its initial concentration and multiplying the result by 100%.

### Liquid Chromatography-Mass Spectrometry

The LC-MS/MS analysis was carried out using the UltiMate 3000 RSLC chromatographic system from Dionex (Sunnyvale, CA, USA) connected with the API 4000 QTRAP triple quadrupole mass spectrometer from Applied Biosystems-Sciex (Foster City, CA, USA). Five microliters of the sample was injected into a C18 Hypersil GOLD 100 mm × 2.1 mm × 1.9 μm with 0.2-μm glass prefilter. The mobile phase used for the analysis was 5 mmol/L of ammonium acetate in water and methanol at a flow rate of 0.2 mL/min. The analytical gradient used for the analysis of bisphenols was as follows: from 70 to 100% methanol in 2 min, 100% methanol for 1 min. Chromatographic column effluent was directed to the mass spectrometer through the electrospray ionization source, the source operated in the negative ion mode. Detection was performed in the following conditions of the ion source and mass spectrometer: curtain gas, 15 psi; nebulizer gas, 40 psi; auxiliary gas, 45 psi; temperature, 450 °C; ion spray voltage, − 4500 V; and collision gas, set to medium. The detected mass transitions and specific parameters of each analyte were summarized (Table 1).

**Table 1:** Parameters of mass spectrometric detection characteristic of the particular analytes

Analyte	Abbreviation	DP (V)	Multiple reaction monitoring transitions (deprotonated molecule [M-H]^−^*m*/*z* → fragment ion *m*/*z*)					
			Analytical	CE (eV)	CXP (V)	Confirmatory	CE (eV)	CXP (V)
Bisphenol A	BPA	− 85	227 → 212	− 27	− 3	227 → 133	− 37	− 10
Bisphenol B	BPB	− 70	241 → 212	− 27	− 3	241 → 147	− 37	− 8
Bisphenol S	BPS	− 70	249 → 108	− 38	− 4	249 → 92	− 50	− 6
Bisphenol E	BPE	− 60	213 → 198	− 25	− 2	213 → 119	− 36	− 9
Bisphenol F	BPF	− 85	199 → 93	− 31	− 6	199 → 123	− 29	− 9
Bisphenol AF	BPAF	− 75	335 → 265	− 30	− 3	335 → 315	− 29	− 6

*DP* declustering potential, *CE* collision energy, *CXP* collision cell exit potential

### Metabolic Activity Measurements

The next stage of the research was devoted to evaluation of the metabolic activity during biodegradation. The metabolic activity of microorganisms was measured using the MTT assay [20], which evaluates the activity of respiratory enzymes. These enzymes convert the yellow MTT to its violet formazan, which is determined spectrophotometrically. The measurements were conducted as follows: every 7 days, the cultures were shaken and 0.9-mL aliquot was collected and mixed in Eppendorf tubes (1.5 mL) with 0.1 mL of freshly prepared 5 mg/mL MTT solution. Thereafter, the samples were incubated 2 h at 22 °C and then centrifuged for 5 min at 15,000*g*. Successively, the supernatant was removed, and the violet precipitate was dissolved in 1 mL of propan-2-ol. The prepared samples were shaken and centrifuged again for 5 min at 5000*g* and the absorbance of the supernatant was measured at 560 nm. The amount of the produced violet formazan dye proportional to metabolic activity was calculated using the extinction coefficient *ε* = 13 1/mM cm.

### Statistical Analysis

The metabolic activity measurements were conducted in three repetitions, and their mean value and standard deviation were calculated. The correlation of the results obtained from the metabolic activity measurements and biodegradation tests was examined using the Microsoft Excel spreadsheet. Because the metabolic activity varied considerably during the biodegradation tests, for each type of inoculum, the dependence between the two sets of results was verified based on both average metabolic activity vs. biodegradation and maximum metabolic activity vs. biodegradation.

## Results and Discussion

**Fig. 2:**
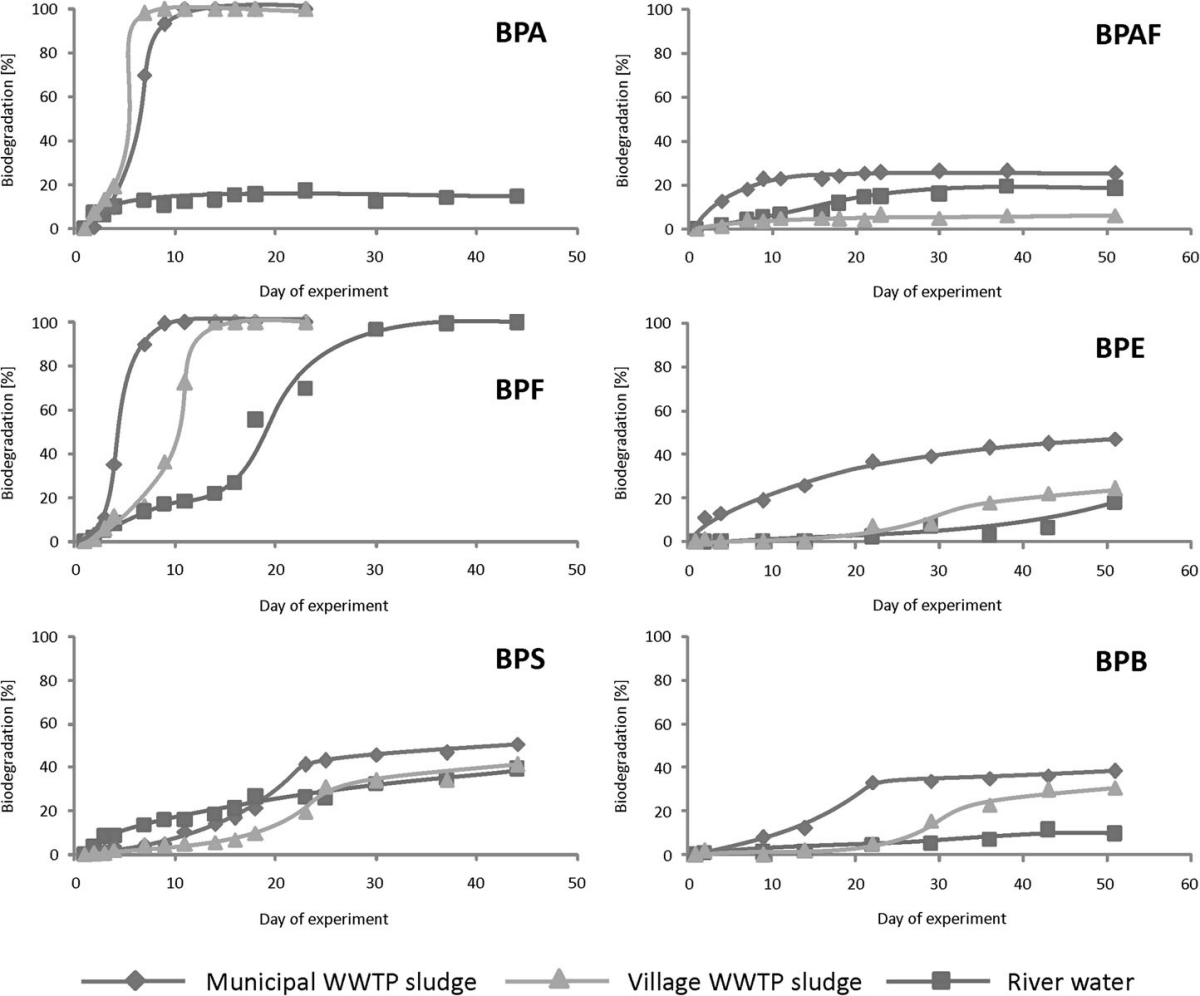
Biodegradation of bisphenols in three types of tests

The test with BPA revealed relatively rapid primary biodegradation of this compound when activated sludge was used as an inoculum (Fig. 2). These results are in accordance with the literature reports showing that primary biodegradation of BPA takes place in less than 5 days in water of different origin, including ship channel water, bayou water, and chemical plant treated process effluent [21]. There was also no considerable difference between the results obtained using activated sludge from the municipal and rural WWTPs. However, biodegradation in the river water was much less and did not exceed 20%. Literature reports often contain opposite results. For example, Klecka et al. [22] reported rapid biodegradation of BPA in river water from seven different locations in the USA and Europe, Danzl et al. [23] reported degradation of BPA in seawater, and Dorn et al. [21] reported degradation of BPA in other types of surface waters (e.g., ship channel water, bayou water) but little (18%) BPA degradation in seawater was also reported [24]. There may be two reasons for less biodegradation of BPA observed in the present study: lesser amounts of bacteria present in the river water than in the media inoculated with activated sludge from the two WWTPs or less biodiversity of bacteria from the river water used in the test. The second reason can be explained by taking into account previous studies indicating greater biodegradation of BPA in the presence of two or more bacterial strains than in the presence of only one bacterial strain [24, 25].

Primary biodegradation of BPF was different from that for BPA (Fig. 2). Similarly, rapid degradation of BPF and BPA was noted in the test using inoculum from a municipal WWTP. Usage of inoculum from a rural WWTP led to slower biodegradation of BPF than BPA. Conversely, in the test with river water, it was possible to achieve 100% primary biodegradation of BPF in 37 days, while degradation of BPA reached less than 20%. These differences in the biodegradation of BPA and BPF illustrate that different bacterial strains are responsible for their removal even though the compounds have similar structures. This confirms previous findings reporting that strains capable of BPA degradation may not degrade BPF [24] and, on the other hand, some bacterial consortia degrade BPF better than BPA [23].

The biodegradation test with BPS (Fig. 2) found considerably less removal of this compound, in the range from 40 to 50%, which was similar in all three tests. The degradation for the two tests with activated sludge is considerably less than that obtained for either BPA or BPF. The biodegradation of BPS in the river water was less than one-half that of BPF but simultaneously two times greater than that of BPA (Fig. 2). Previous investigations also found problems with the biodegradation of BPS [23, 24]. However, bacteria capable of BPS biodegradation also exist [26].

Primary biodegradation of BPAF, BPE, and BPB was minimal in all tests (Fig. 2) with the least degradation found for BPAF containing fluorinated carbon atoms in the ring-linking group. Problems with the biodegradation of these compounds under aerobic conditions may result from different mechanisms involved in primary degradation of bisphenols. Degradation of BPS begins with the oxidation of its rings [26] and degradation of BPA and BPF is initiated in their ring-linking groups although there are different mechanisms involved in the primary degradation of these two compounds [25, 27]. Therefore, bacteria capable of degrading one bisphenol do not necessarily degrade other bisphenols which is in accordance with the results of the present study as well as previously published studies [24].

As presented above and in previous studies of other authors, BPA can undergo considerable primary biodegradation [21–23]. Nevertheless, it was also reported [28] that consequential amounts of BPA continue to be released from WWTPs. Between 57.5 and 257.0 μg/L, BPA were measured in effluents from 5 different WWTPs, and removal efficiency of BPA ranged from 22.67 to 86.32% [28]. Therefore, the presence of BPA (as well as other bisphenols) in the environment is still a great problem [29–31]. Minimal biodegradation of some potential candidates for the replacement of BPA evaluated in the present study should turn our attention to their monitoring after BPA usage is banned in many countries.

**Fig. 3:**
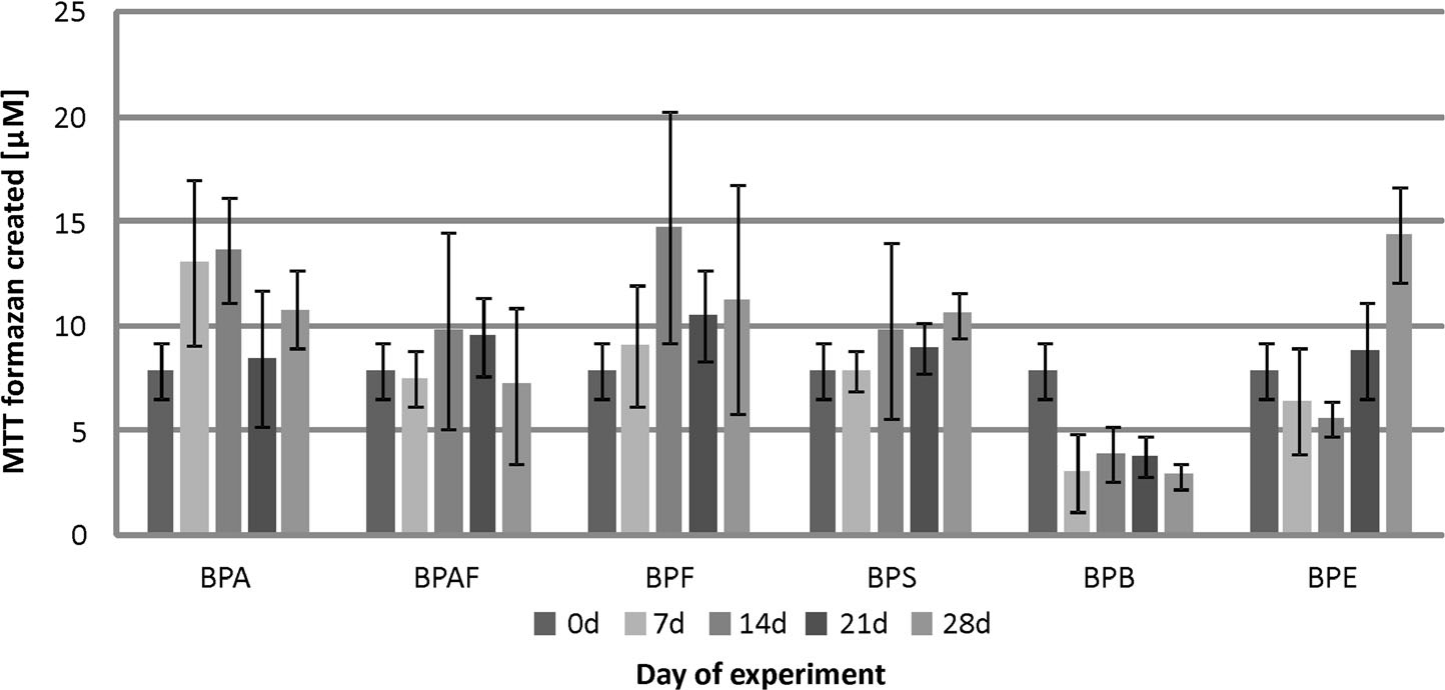
Metabolic activity of the river water microbiota during the biodegradation of bisphenols

Additional important information was obtained from the results of metabolic activity measurements of microorganism consortia during the biodegradation. The microbiota from the river water exhibits the least initial metabolic activity (corresponding to no more than 10 μM of MTT formazan), compared with the metabolic activity of WWTP activated sludge microorganisms (reaching nearly 150 μM of MTT formazan for samples from the rural WWTP). Among investigated compounds, the strongest impact on river microbiota was displayed by BPB, which minimized the metabolic activity to 3 μM of MTT formazan after only 7 days (Fig. 3). In the case of other chemical compounds, the values of metabolic activity oscillated over time around the original value. Only in the presence of BPE was a marked increase in the metabolic activity of cells observed.

**Fig. 4:**
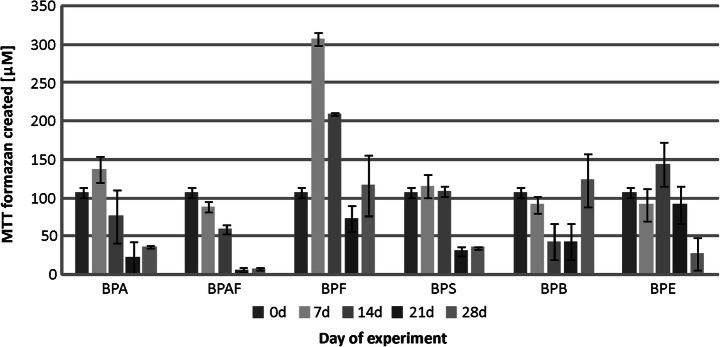
Metabolic activity of the municipal WWTP activated sludge microbiota during the biodegradation of bisphenols

**Fig. 5:**
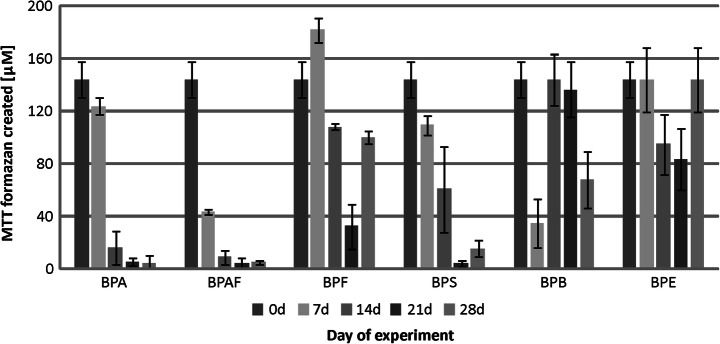
Metabolic activity of the rural WWTP activated sludge microbiota during the biodegradation of bisphenols

The results obtained for the municipal WWTP activated sludge revealed that BPA, BPAF, BPS, and BPE clearly reduced the metabolic activity of microorganisms (Fig. 4). The strongest decline was found for BPAF. In cultures containing this compound, after 21 days, the metabolic activity did not exceed 10 μM MTT formazan. It should be emphasized that in both cultures with BPF and BPB, after 4 weeks, metabolic activity returned to its initial value. Importantly, during the biodegradation of BPF, a threefold increase in the metabolic activity was observed. The microorganisms present in the activated sludge from the rural WWTP proved to be sensitive to bisphenol compounds. As with the active sediment from the municipal WWTP, the strongest reduction in the metabolic activity of microorganisms was observed for cultures with BPA, BPAF, and BPS (Fig. 5). In these samples, after 21 days, the metabolic activity did not exceed 5% of its initial value. It should be emphasized that also for this microbiome, after 7 days of experimentation, an increase was found in metabolic activity of microorganisms in culture with BPF, similar to that for the municipal WWTP activated sludge. In turn, the presence of BPE did not significantly alter the cellular metabolic activity. Moreover, fluctuations of the parameter determined during biodegradation of BPB indicated dynamic processes occurring in these cultures.

As it was expected that biodegradation could be connected with increased metabolic activity, tests verifying such a potential relationship were performed. The varying metabolic activity, especially in experiments where it dropped substantially from the initial value, forced a decision on testing two different sets of results. Therefore, both average metabolic activity vs. biodegradation and maximum metabolic activity vs. biodegradation were tested. For the tests on correlation of the average metabolic activity vs. biodegradation, the coefficient of determination was only 0.3118, 0.4103, and 0.0150 for river water, municipal WWTP, and rural WWTP inocula, respectively. Similarly, for the tests on the correlation of the maximum metabolic activity vs. biodegradation, the coefficient of determination was also low, i.e., 0.2606, 0.4449, and 0.3636 for river water, municipal WWTP, and rural WWTP inocula, respectively. These results clearly indicate that the high activity is not correlated with elevated biodegradation. BPA, which was effectively degraded by activated sludge microorganisms from both WWTPs, strongly decreased metabolic activity in the samples. In contrast, equally biodegradable BPF (like BPA) even caused some increase of the metabolic activity. On the other hand, reducing metabolic activity for the tests with BPS and BPAF is similar to that caused by BPA, but their degradation is still much less. The considerable difference observed for reducing metabolic activity can be related to two different root causes. First, it can be decreased because of the depletion of nutrients (for BPA). Secondly, it can be connected with harmful effects of bisphenols on bacteria or simply lack of nutrients available to bacterial species (for BPS and BPAF). However, these assumptions could not be supported by the obtained results.

The changes in metabolic activity during pollutant degradation have not been studied before. The difference in microorganism activity occurring during the biodegradation may be a result of complex changes in the composition of microbial consortium. As has been observed previously [32], tetrachlorobisphenol A noticeably modified the microbiome leading to increased abundance of *Proteobacteria* as well as a decrease in *Acidobacteria* and *Parcubacteria* share in the analyzed activated sludge. What is more, changes in composition of consortia have been revealed during degradation of bisphenol A [33]. Moreover, the specific substrate can strongly regulate the metabolic activity in the cells of a single strain as has been reported [34]. The synergistic effect of these two main factors results in the observed changes in the efficiency of biodegradation.

## Conclusions

During the tests, it was found that only BPA and BPF undergo complete primary biodegradation supporting the possibility of removal of these compounds from the environment. Other bisphenols were poorly biodegraded even though BPB and BPE share similar structures with the alkyl linking group and no additional sulfur or fluorine atoms. This illustrates great specialization of bacteria degrading particular bisphenols which is in accordance with previously published studies [22]. Species degrading one bisphenol do not degrade all the other bisphenols. Thus, simple replacement of legally regulated BPA with other bisphenols may result in increased contamination of the environment at least until bacteria adapt to new nutrients.

An important conclusion resulting from the comparison of the results from biodegradation and metabolic activity is that elevated metabolic activity is not directly correlated with the efficiency of biodegradation. Decline of activity is visible in tests with elevated as well as minimal biodegradation results. Surely, different phenomena are responsible for depressed activity with BPA vs. BPAF or BPS. It must be emphasized that for many compounds including the tested bisphenols, changes of microbial consortia may be induced. The bacterial species dominating after primary biodegradation may not be capable of further degradation. Thus, for BPA, decline of metabolic activity can be associated with the depletion of nutrients, while for BPAF and BPS, both their harmfulness to bacteria and their absence of nutritional use by bacteria result in no degradation of BPAF and BPS, even primary biodegradation.
